# Enhancement of radiation response with bevacizumab

**DOI:** 10.1186/1756-9966-31-37

**Published:** 2012-04-26

**Authors:** Tien Hoang, Shyhmin Huang, Eric Armstrong, Jens C Eickhoff, Paul M Harari

**Affiliations:** 1 Department of Medicine, University of Wisconsin-Madison, Madison, WI, USA; 2 Department of Human Oncology, University of Wisconsin-Madison, Madison, WI, USA; 3 Department of Biostatistics and Medical Informatics, University of Wisconsin-Madison, Madison, WI, USA

**Keywords:** Anti-angiogenesis, VEGF, Bevacizumab, Radiation

## Abstract

**Background:**

Vascular endothelial growth factor (VEGF) plays a critical role in tumor angiogenesis. Bevacizumab is a humanized monoclonal antibody that neutralizes VEGF. We examined the impact on radiation response by blocking VEGF signaling with bevacizumab.

**Methods:**

Human umbilical vein endothelial cell (HUVEC) growth inhibition and apoptosis were examined by crystal violet assay and flow cytometry, respectively. *In vitro* HUVEC tube formation and *in vivo* Matrigel assays were performed to assess the anti-angiogenic effect. Finally, a series of experiments of growth inhibition on head and neck (H&N) SCC1 and lung H226 tumor xenograft models were conducted to evaluate the impact of bevacizumab on radiation response in concurrent as well as sequential therapy.

**Results:**

The anti-angiogenic effect of bevacizumab appeared to derive not only from inhibition of endothelial cell growth (40%) but also by interfering with endothelial cell function including mobility, cell-to-cell interaction and the ability to form capillaries as reflected by tube formation. In cell culture, bevacizumab induced a 2 ~ 3 fold increase in endothelial cell apoptosis following radiation. In both SCC1 and H226 xenograft models, the concurrent administration of bevacizumab and radiation reduced tumor blood vessel formation and inhibited tumor growth compared to either modality alone. We observed a siginificant tumor reduction in mice receiving the combination of bevacizumab and radiation in comparison to mice treated with bevacizumab or radiation alone. We investigated the impact of bevacizumab and radiation treatment sequence on tumor response. In the SCC1 model, tumor response was strongest with radiation followed by bevacizumab with less sequence impact observed in the H226 model.

**Conclusions:**

Overall, these data demonstrate enhanced tumor response when bevacizumab is combined with radiation, supporting the emerging clinical investigations that are combining anti-angiogenic therapies with radiation.

## Background

Tumor angiogenesis is critical for tumors to grow and spread. Four decades ago, Folkman proposed targeting the tumor vasculature as a strategy to treat cancer [1]. Since then advances in biology have provided new tools and knowledge in the area of angiogenesis. A key discovery was the identification of vascular endothelial growth factor (VEGF), a key angiogenic protein critical for the growth of endothelial cells and development of tumor blood vessels [2-4]. VEGF herein emerged as an attractive target for anticancer therapy. It has been demonstrated in animal models that neutralization VEGF could inhibit the growth of primary tumor and metastases. In small 1–2 mm foci of tumor cells, blocking the VEGF pathway inhibited the “angiogenic switch”, i.e. preventing tumor transformation from an avascular to vascular phase, thus maintaining a quiescent state [5]. Bevacizumab is a humanized monoclonal antibody which neutralizes the VEGF ligand. Since its development in the late 1990s, the anti-tumor effects of this anti-VEGF antibody have been studied in various preclinical cancer models [6] as well as in clinical trials. The combination of bevacizumab and cytotoxic chemotherapy prolongs survival in patients with advanced colorectal, lung or breast cancer. Bevacizumab is currently approved for use in combination with chemotherapy in those diseases, as well as monotherapy in recurrent glioblastoma.

Another potential treatment strategy is to combine bevacizumab with radiation to enhance the therapeutic index. Radiation dose escalation is limited in most anatomic sites by normal tissue toxicities. Therefore, combining radiation with targeted agents such as anti-angiogenic in an effort to augment radiation impact and improve tumor control is desirable. It has been shown that blocking VEGF with recombinant human anti-VEGF antibody can enhance radiation response in preclinical studies [7]. Augmentation of tumor response was also observed when radiation was combined with other anti-angiogenic or vascular disrupting drugs [8-16].

The primary objective of this study was to investigate the anti-angiogenic and anti-tumor activity of bevacizumab in combination with radiation in human endothelial cells as well as in H&N and lung tumor models. We also explored the sequencing treatment of bevacizumab and radiation.

## Methods

### Chemicals, cell lines and animals

Bevacizumab was provided by Genentech (South San Francisco, CA). SCC1, a human head and neck squamous carcinoma cell line was kindly provided by Dr. Tom Carey (University of Michigan). The lung cancer cell line H226 was from the laboratory of Dr. Minna and Dr. Gazdar (University of Texas Southwestern Medical School). Supplement of all materials used in our experiments can be found in our previous publication [[15]].

### HUVEC growth inhibition assay

In this crystal violet assay, growing HUVEC seeded in 6-well plates (50,000 cells/well) were treated with bevacizumab in EGM-2 at various concentrations (0–10 μM). After 3 days, cells were stained with crystal violet. The method of this assay was described in detail in previous publication [15]. The relative percentage of cell growth was calculated by comparison between the bevacizumab-treated and control wells.

### Flow cytometry analysis of HUVEC apoptosis

Growing HUVEC were treated with EGM-2 (control), bevacizumab 0.1 μM, radiation 6 Gy, or combined bevacizumab and radiation. After 24 and 48 hours of incubation, cells were harvested, prepared, and stained with propidium iodide (PI) prior to flow cytometry analysis. The procedure was described in detail in previous publication [15]. DNA distributions were analyzed by Modfit for the proportion of apoptotic cells.

### *In vitro* angiogenesis (HUVEC tube formation) assay

In this assay, HUVEC (40,000 cells) were seeded atop of matrigel membrane in the absence (control) or presence of bevacizumab (0.5 μM and 5 μM). The method of this assay was described in detail in previous publication [15]. The plate was examined and photographed for the formation of capillary-like endotubes under a phase-contrast microscopy at 3 h, 6 h and 22 h.

### *In vivo* angiogenesis (matrigel plug) assay

The method of this assay was described in detail in previous publication [15]. In brief, 4 groups of mice with H226-containing matrigel plugs were treated with IgG (control), bevacizumab alone (1 mg/kg intraperitoneally), radiation alone (2 Gy/fraction), or combination treatment in which bevacizumab was administered immediately following radiation, twice a week for 2 weeks. At the end of week 2, mice were injected with FITC-Dextran solution. The plugs were removed and examined for the perfused blood vessels. The intensity of fluorescence in captured images was quantified by Adobe Photoshop software.

### Growth inhibition assay in tumor xenograft models

A series of *in vivo* experiments in athymic mice bearing SCC1 and H226 xenografts were conducted to examine the anti-tumor activity of bevacizumab, radiation and combined therapy in concurrent and sequential fashion. Design and treatment schedule of those experiments are described in the Results Section. Details on xenografts, animal care, tumor measurement and radiation delivery were described in previous publication [15].

### Statistical analysis

Analysis of variance (ANOVA) was performed to compare tumor volume in groups of mice treated with bevacizumab and/or radiation with the control group. Treatment interaction and linear contrasts were used to evaluate the synergistic effect of the bevacizumab and radiation therapy combination. Tumor volume was log-transformed to meet the assumption of normality. Effects of bevacizumab and radiation on tumor growth in mice bearing SCC1 and H226 xenografts were analyzed using ANOVA and linear mixed-effects models. An autoregressive correlation structure was assumed to account for correlations between repeated measurements within an experimental unit. Tukey’s HSD method was used to control the type 1 error for the pairwise comparisons between treatment groups. All *p* values were two sided and considered significant when ≤0.05. Statistical analyses were performed with SAS statistical software (version 8.2; SAS Institute, Cary, NC).

## Results

### Bevacizumab inhibits HUVEC proliferation *in vitro* and tumor growth *in vivo*

**Figure 1:**
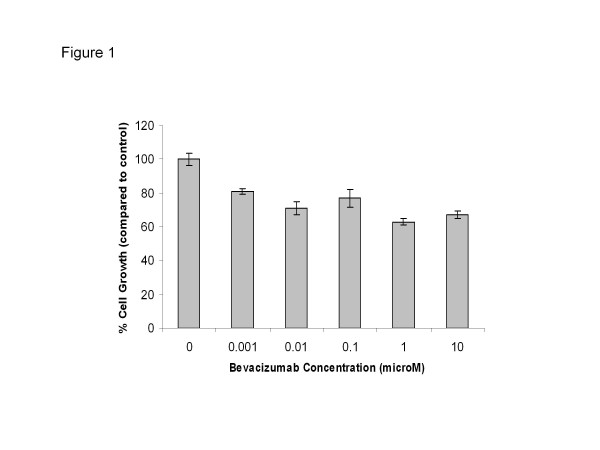
Inhibitory effect of bevacizumab on proliferation of HUVEC.

In the crystal violet assay, bevacizumab induced a modest inhibition of HUVEC growth at a concentration as low as 0.001 μM (Figure 1). This inhibition was observed across 5 logs of bevacizumab dose ranging from 0.001Â μM - 10Â μM with approximately 30-40% HUVEC growth inhibition. No clear dose response effect was observed suggesting saturation of VEGF blockade in the higher dose range.

**Figure 2:**
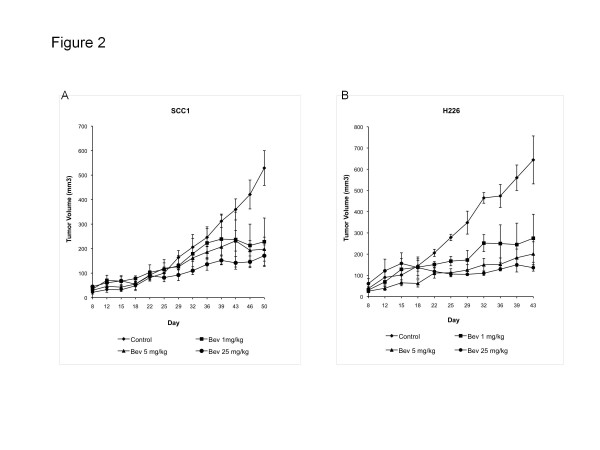
**Inhibitory effect of bevacizumab on tumor growth in SCC1 (A) and H226 (B) xenograft models.** Four groups of mice (n = 3 tumors per treatment group for each cell line) were treated with: IgG (control), bevacizumab 1 mg/kg, 5 mg/kg and 25 mg/kg. *Bev, bevacizumab.*

In the first animal experiment designed to test the anti-tumor activity of bevacizumab in our xenograft models, mice with SCC1 and H226 xenografts (n = 3 per treatment group for each cell line) were treated with IgG (control) or 3 dose levels of bevacizumab (1, 5 and 25 mg/kg i.p.) twice weekly for a total of 9 doses (Figure 2A and B). Compared with controls, bevacizumab at all 3 doses significantly inhibited tumor growth in both SCC1 (p values of 0.04, 0.05, and 0.03, respectively) and H226 groups (p values of 0.06, 0.04, and 0.01). There was no significant statistical difference in anti-tumor activity observed among the three bevacizumab groups. This result is consistent with other reports demonstrating the maximal inhibitory activity of bevacizumab in tumor xenograft models at approximately 1–2 mg/kg [6]. Based on this result, a dose of 0.75-1 mg/kg of bevacizumab was chosen for subsequent experiments to investigate the combination of bevacizumab and radiation.

### Bevacizumab inhibits the formation of HUVEC capillary-like network

**Figure 3:**
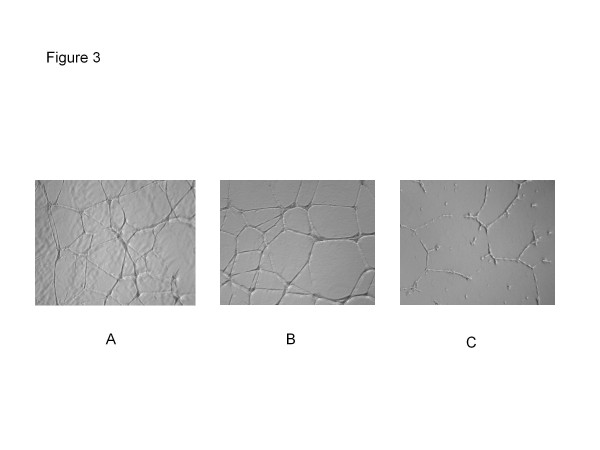
**Inhibitory effect of bevacizumab on HUVEC capillary-like network formation following 22 hours of treatment:** (**A**) IgG (control), (**B**) Bevacizumab 0.5 μM, and (**C**) Bevacizumab 5 μM.

In the tube formation assay, we observed a quick attachment of HUVEC onto the matrigel in the control wells. Indeed, cells mobilized on the gel, spread out and generated lateral processes to form intercellular connections within 3 hours of seeding, with a network of endotubes well established by 6 hours. This capillary-like network was well maintained after 22 hours in the control wells (Figure 3A). In the 0.5 μM bevacizumab wells, little inhibitory effect was observed (Figure 3B). However, bevacizumab at 5 μM clearly prevented the mobilization and generation of lateral processes of HUVECs with only fragmented tubes being seen (Figure 3C). As seen in the figures, the total numbers of intact endotubes in the control, bevacizumab 0.5 μM and bevacizumab 5 μM groups at 22 hours of incubation are 42, 39, and 0, respectively. This result suggests that bevacizumab inhibits not only HUVEC growth but also endothelial cell function.

### Bevacizumab enhanced radiation-induced apoptosis in HUVEC

**Figure 4:**
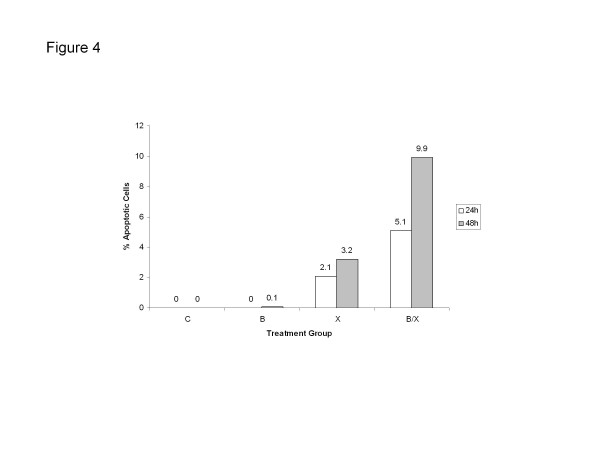
**Effect of bevacizumab with and without radiation on HUVEC apoptosis.** Four groups of HUVEC were treated with: IgG (control), bevacizumab (**B**), radiation (**X**), and combined bevacizumab and radiation (B/X).

To investigate the apoptotic effect of radiation and bevacizumab, we treated HUVEC with bevacizumab, radiation, or both (Figure 4). Apoptosis was observed in cells treated with radiation alone and combined radiation and bevacizumab, but not in the control or bevacizumab alone group. Moreover, this experiment demonstrated the ability of bevacizumab to enhance radiation-induced apoptosis in HUVEC, with 5.1% and 9.9% of cells treated with combined therapy undergoing apoptosis after 24 and 48 hours respectively versus 2.1% and 3.2% in cells treated with radiation alone.

### Concurrent administration of bevacizumab and radiation inhibits *in vivo* tumor vascularization

**Figure 5:**
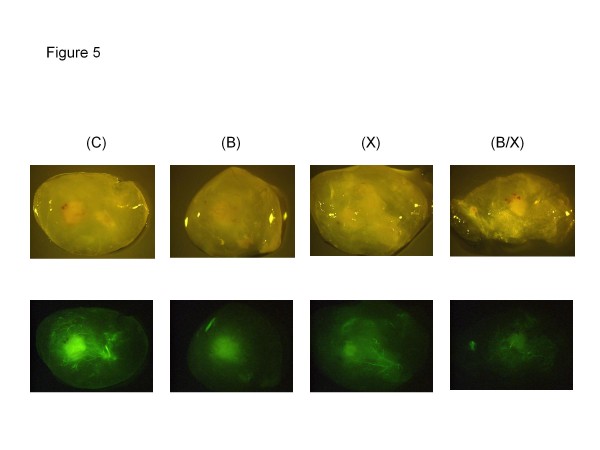
**Activity of bevacizumab with and without radiation on blood vessel formation in tumor xenograft models.** Four groups of mice with H226 tumors in Matrigel plugs were treated with: IgG (control), bevacizumab (**B**), radiation (**X**), and combined bevacizumab and radiation (**B/X**). Pictures depict the matrigel plugs with visible tumors and blood vessels (green signal of FITC-Dextran).

To investigate the anti-angiogenic effect of bevacizumab in combination with radiation, we performed an *in vivo* angiogenesis assay in 4 groups of mice with H226 tumor xenografts growing in matrigel plugs (Figure 5): control IgG, bevacizumab alone (1 mg/kg twice a week x 4 doses), radiation alone of 8 Gy (2 Gy/fraction twice a week x 4 doses), and concurrent bevacizumab and radiation. There was a reduction of tumor blood vessels observed in mice treated with either bevacizumab or radiation alone. However, the greatest reduction in tumor vascularization was observed in animals receiving both bevacizumab and radiation. The mean quantitative fluorescence of the tumor vasculature was significantly lower in the combined treatment group (22.9) in comparison to bevacizumab alone (34.8), radiation alone (35.2), and control group (47). This experiment suggested a synergistic interaction between bevacizumab and radiation (p = 0.0054).

### Bevacizumab augments tumor response to radiation

**Figure 6:**
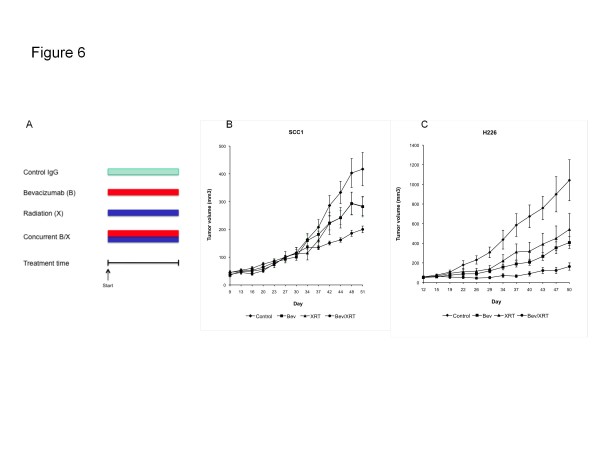
**Anti-tumor activity of bevacizumab and radiation given concurrently in SCC1 and H226 xenograft models.** Four groups of mice with SCC1 and H226 tumors were treated with: IgG (control), bevacizumab (**B**), radiation (**X**), and concurrent bevacizumab and radiation (B/X). (**A**) Treatment schedule, and tumor growth inhibition in (**B**) SCC1 and (**C**) H226 models (n = 8 tumors per treatment group for each cell line).

In this experiment, four groups of mice bearing SCC1 or H226 xenografts (n = 8 tumors/treatment group/cell line) were treated with: control IgG, bevacizumab alone (1 mg/kg twice a week), radiation alone (twice a week with 2.5 Gy/fraction in SCC1 and 2 Gy/fraction in H226 models), or concurrent bevacizumab and radiation (Figure 6A). The SCC1 and H226 groups were treated for 4.5 weeks (9 treatments with a total irradiation dose of 22.5 Gy) and 2 weeks (4 treatments with a total dose of 8 Gy), respectively. The irradiation dose and treatment schedule was chosen based on our previous experience with the two cancer models. We have observed that the H226 xenograft model is significantly more sensitive to the anti-tumor effect from radiation than the SCC1 model. The results demonstrated that monotherapy with either bevacizumab or radiation inhibited tumor growth (Figure 6B and C). However, the strongest inhibitory effect was observed with the concurrent administration of bevacizumab and radiation.

### Impact of treatment sequence of bevacizumab and radiation

**Figure 7:**
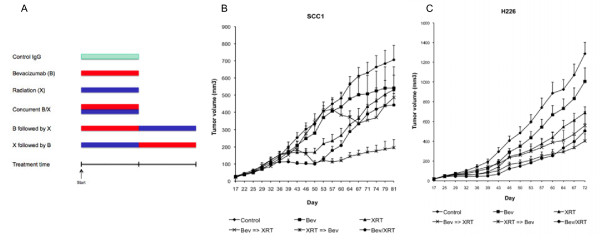
**Impact of treatment sequence with bevacizumab and radiation.** Six groups of mice with SCC1 and H226 tumors were treated with: IgG (control), bevacizumab (**B**), radiation (**X**), concurrent bevacizumab and radiation (B/X), bevacizumab followed by radiation (B⇒X), and radiation followed by bevacizumab (X⇒B). (**A**) Treatment schedule, and tumor growth inhibition in (**B**) SCC1 and (**C**) H226 models (n = 16 tumors per treatment group for each cell line).

This experiment involved 6 groups of mice bearing H226 or SCC1 tumors: (n = 16 tumors per treatment group for each cell line) [1] Control IgG, [2] Bevacizumab (0.75 mg/kg twice a week), [3] Radiation (twice a week with 2.5 Gy/fraction in SCC1 and 2 Gy/fraction in H226 models), [4] Concurrent bevacizumab and radiation, [5] Bevacizumab followed by radiation, and [6] Radiation followed by bevacizumab (Figure 7A). The duration of bevacizumab or radiation treatment was 2.5 weeks (SCC1) and 1.5 weeks (H226). The total irradiation dose was 12.5 Gy (SCC1) and 6 Gy (H226). In the sequential therapy groups, animals completed a course of either bevacizumab or radiation before switching to the other therapy. The purpose of this experiment is to evaluate the impact of treatment sequence of bevacizumab and radiation (groups 4, 5 and 6). There was an increase in tumor inhibition with combined regimens in concurrent or sequential fashion compared to monotherapy. Furthermore, among the three SCC1 combination therapy groups, it appeared that tumor response was strongest with radiation followed by bevacizumab (Figure 7B). By day 81, tumors in this group had a mean tumor volume < 200 mm^3^, while tumors in the other two combined treatment groups regrew (> 400 mm^3^) after a period of response. This impact of treatment sequence on tumor response was not observed in the H226 experiment, with no significant difference in anti-tumor activity seen within the three combined treatment groups (Figure 7C).

## Discussion

In this current study, we confirm the ability of the anti-VEGF monoclonal antibody bevacizumab to inhibit endothelial cell proliferation and disrupt the formation of capillary-like networks in culture. In the H&N and lung cancer xenograft models, treatment with bevacizumab inhibited tumor vascularization and inhibited volume growth of both SCC1 and H226 tumors. However, the growth inhibitory effect of bevacizumab is not complete, suggesting the potential value of combining bevacizumab with other cytotoxic modalities, such as radiation to achieve more potent therapeutic effects.

In this work, we demonstrate that radiation combined with bevacizumab reduced the formation of tumor vasculature and inhibited tumor growth in SCC1 and H226 cancer xenograft models more strongly than either modality alone (Figure 6). This is consistent with prior work using the recombinant human monoclonal anti-VEGF 165 antibody in mouse models bearing other human cancers [7]. Our findings confirm that neutralizing the VEGF ligand with bevacizumab can augment tumor response to radiation. Works from our laboratory and others have previously demonstrated that radiation response is enhanced by blocking the VEGF signaling pathway using small molecule VEGF receptor tyrosine kinase inhibitors such as ZD6474 [11], SU6668 [12] and PTK787/ZK222584 [13], or by directly targeting tumor blood vessels with vascular targeting agents such as ZD6126 [14,15] and combretastatin [16].

The anti-tumor effect of this combination approach is consistent with the two-compartment model described by Folkman [17]. According to this model, tumors are comprised of distinct compartments including tumor cells and endothelial cells. By targeting the endothelial cell compartment, bevacizumab not only inhibits the supply of oxygen and nutrients to the tumor, but also interrupts the “paracrine effect” by inhibiting endothelial secretion of growth factors such as IGF1, bFGF, and HB-EGF, which can stimulate tumor proliferation. In parallel, by targeting the tumor compartment, radiation kills cancer cells and thereby shuts down their production of “pro-angiogenic” factors, thus indirectly affecting the endothelial compartment. We have also observed that treatment with radiation can inhibit endothelial cell proliferation and stimulate apoptosis [15] and G2/M arrest (nonpublished data), suggesting direct inhibitory effects of radiation on this compartment.

A current question of interest in clinical trial design regards the optimal sequencing of radiation and anti-angiogenic drugs to achieve maximal benefit. A valid concern is whether targeting the tumor vasculature will decrease tumor blood perfusion, resulting in tumor hypoxia, and thereby diminishing the effects of radiation. To investigate the impact of treatment sequencing on tumor response, we designed sequence experiments as described in Figure 7. In the SCC-1 model, it appeared that tumor control was best achieved with the regimen of radiation followed by bevacizumab. This result supports the hypothesis that hypoxia induced by bevacizumab may hinder radiation effect. However, we found no clear difference between sequence regimens in the H226 tumors.

Consistent with our observation in the SCC-1 tumors, preclinical studies have shown that delivering ZD6126 prior to radiation to U87 glioblastoma xenografts resulted in acute drop in tumor oxygen tension and attenuation of the killing effects of radiation [18]. Further, in KHT sarcoma models, the strongest anti-tumor activity was achieved when ZD6126 was administered one hour following radiation [14]. These observations suggest a negative impact of ZD6126-induced hypoxia on radiation effect. However, the concept of normalization of tumor vasculature proposed by Jain *et al.* supports a strategy of using anti-angiogenic drugs to improve efficacy of radiation [19]. This theory suggests that short term treatment of anti-angiogenic agents may “normalize” the network of abundant but chaotic, leaky and dysfunctional tumor vasculature, thus restore the integrity and function of the blood vessels, leading to a decrease in interstitial fluid pressure and the improvement in tumor oxygenation [19]. Therefore, this process of vascular normalization could enhance the tumor killing activity of radiation as well as improve drug delivery into the tumor [19]. Although the induction of vascular normalization by anti-angiogenic agents has been supported by preclinical studies [20], it remains a challenge to capture the transient “tumor oxygenation window” for the delivery of radiation. We are commencing real-time imaging of tumor hypoxia profiles in animals during treatment to help explore optimal strategies for this combined therapy.

In the clinic, several clinical phase I/II studies have been conducted to investigate the safety and efficacy of radiation and bevacizumab in cancer patients. The first report came from a series of 6 patients with locally advanced rectal carcinoma who were treated in a phase I trial with induction therapy of bevacizumab (5 mg/kg x 1 dose) followed by radiation in combination with bevacizumab and 5-fluorouracil, then surgical resection [21]. This pilot study demonstrated that a single dose of bevacizumab induction lead to a significant decrease in interstitial fluid pressure, tumor blood perfusion, and microvascular density on day 12 [21]. The subsequent phase II trial in the same patient population demonstrated that bevacizumab induction therapy followed by concurrent bevacizumab and chemoradiation appeared safe and active with a 5-year local control and overall survival of 100% [22]. The combination of bevacizumab with radiation was also investigated in early clinical studies in other diseases including pancreatic cancer [23] and head and neck cancer [24], in which bevacizumab was started either prior or concurrently with chemoradiation.

## Conclusions

In conclusion, the current study demonstrates enhanced tumor response when bevacizumab is combined with radiation. These data support the strategy of blocking the VEGF signaling pathway and targeting tumor blood vessels to improve the therapeutic index of radiation. Important questions remain including optimization of modality sequencing to achieve best outcome. Further molecular and genetic knowledge regarding angiogenesis, interaction between radiation and tumor, blood vessels as well as microenvironment are needed. New imaging tools that capture real time changes in tumor oxygenation may provide further guidance regarding optimal sequencing of combined antiangiogenic therapies and radiation. Further studies of anti-angiogenic drugs and irradiation in non-squamous carcinoma lung and squamous carcinoma H&N models are warranted.

## Abbreviations

VEGF: Vascular endothelial growth factor; HUVEC: Human umbilical vein endothelial cell; H&N: Head and neck; Bev: Bevacizumab; XRT: Irradiation.

## Competing interests

Dr. Paul M. Harari received research funding from NCI/NIH and Genentech Inc (paid to the University of Wisconsin) as well as patents and royalties (paid to Dr. Harari and the Wisconsin Alumni Research Foundation). Other authors do not have conflict of interest.

## Authors’ contributions

TH participated in the design of the study, carried out experiments, performed data analysis, and drafted the manuscript. SH participated in the design of the study, assisted in xenograft experiments and data analysis, and edited the manuscript draft. EA participated in the design of the study, assisted in experiments, data analysis and manuscript draft. JCE performed statistical analysis, assisted in data analysis and manuscript draft. PMH participated in the design of the study, performed data analysis, and edited the manuscript draft. All authors read and approved the final manuscript.
